# Occurrence of metabolic syndrome in midlife in relation to cardiovascular morbidity and all-cause mortality—lessons from a population-based matched cohort study with 27 years follow-up

**DOI:** 10.1136/bmjopen-2023-081444

**Published:** 2024-09-16

**Authors:** Lena Lönnberg, Jerzy Leppert, John Ohrvik, Mattias Rehn, Abbas Chabok, Mattias Damberg

**Affiliations:** 1 Centre for Clinical Research Vastmanland Hospital Vasteras, Uppsala University, Uppsala, Sweden; 2 Division of Surgery, Danderyd University Hospital, Stockholm, Sweden; 3 Uppsala University Department of Public Health and Caring Sciences, Uppsala, Sweden

**Keywords:** Hypertension, Factor Analysis, Statistical, Primary Health Care, Risk Factors, Diabetes & endocrinology

## Abstract

**Abstract:**

**Objectives:**

We examined how asymptomatic metabolic syndrome (MetS) in midlife affects cardiovascular (CV) morbidity and all-cause mortality later in life and studied difference in time to event and from the individual components related to MetS.

**Design:**

Population-based matched cohort study including data from a screening programme for identification of CV risk factors.

**Setting:**

Primary care, County of Västmanland, Sweden.

**Participants:**

All inhabitants turning 40 or 50 years between 1990 and 1999 were invited to a health screening. Total 34 269 (60.1%) individuals completed the health examination. Participants that met a modified definition of MetS were individually matched to two controls without MetS with regard to age, sex and date of health examination.

**Interventions:**

None.

**Main outcome measures:**

CV events and all-cause mortality from the index examination to June 2022.

**Results:**

All 5084 participants with MetS were matched to two controls. There were 1645 (32.4%) CV events in the MetS group and 2321 (22.8%) CV events for controls. 1317 (25.9%) MetS and 1904 (18.7%) control subjects died. The adjusted HRs (aHR) for CV event and death were significantly higher when MetS was present (aHR) 1.39*** (95% CI 1.28 to 1.50) and 1.27*** (95% CI 1.16 to 1.40) respectively. The factor analysis identified three dominating factors: blood pressure, cholesterol and blood glucose. Mean time for first CV event and death was 2.6 years and 1.5 years shorter respectively for participants within the highest quartile compared with participants with lower mean arterial blood pressure (MAP). The aHR for each 10 mm Hg increased MAP were 1.19*** (95% CI 1.15 to 1.23) for CV event and 1.16*** (95% CI 1.11 to 1.21) for death.

**Conclusion:**

The risk of a CV event and premature death is significantly increased when MetS is present. Early detection of metabolic risk factors, especially, high blood pressure, opens a window of opportunity to introduce preventive treatment to reduce CV morbidity and all-cause mortality.

**STRENGTHS AND LIMITATIONS OF THIS STUDY**

- The underlying health screening programme for this study was large scale and presents 27-year follow-up time.
- The population-based matched cohort study design and factor analysis enabled in-depth studies of the long-term effects of cardiovascular risk factors and blood pressure in more detail.
- The data does not provide information if the screening for cardiovascular disease risk factors induced pharmacological treatment which may hamper the results.
- The definition of metabolic syndrome included total cholesterol only, not high-density lipoprotein and triglycerides, which makes it harder to evaluate the impact of this risk factor.
- Generalisability is limited due to a homogenous population, mainly Caucasian men and women.

## Introduction

 The metabolic syndrome (MetS) is a cluster of common risk factors, including insulin resistance, impaired glucose tolerance, abdominal obesity, dyslipidaemia and hypertension.^1^ Multiple criteria exist to define the metabolic syndrome for example the definitions of The National Cholesterol Education Programme Adult Treatment Panel III^2^ and International Diabetes Federation.^3^ Despite different definitions of MetS, many studies with different populations have shown that when the criteria for MetS are reached the individual is at higher risk for cardiovascular disease (CVD), CVD-related mortality and all-cause mortality.^4^,^9^ Early identification of MetS and its individual components is crucial, that is, before these risk factors are manifested in for example CVD or diabetes mellitus type 2 (DMT2). Identifying asymptomatic MetS opens for a successful management including pharmacological treatment and support for healthy lifestyle choices.

Introducing screening programmes for the identification of cardiovascular risk factors is one example on how this can be performed. The WestmannIa Cardiovascular Risk FacTORs StudY (WICTORY) was a large-scale screening programme directed to the middle-aged population in the county of Västmanland, Sweden, launched in 1990. By using data from this cohort, we were able to follow what impact asymptomatic MetS, as well as its individual components, had on the development of CVD and all-cause mortality up to three decades later.

### Objective

The primary aim of this prospective population-based study was to examine how the occurrence of asymptomatic MetS in 40 and 50 years old individuals affects their CVD morbidity and all-cause mortality later in life and to study difference in time to event between MetS and controls.

The secondary aim was to examine effects from the individual components related to MetS regarding CVD morbidity and all-cause mortality later in life.

### Hypothesis

Hypertension, as one of the components of MetS discovered at the age of 40 and 50, respectively, is a major risk factor for development of CVD and premature death.

## Materials and methods

### Study population

The intention of the screening programme was to offer a free screening for cardiovascular risk factors to all inhabitants in the county of Västmanland the year they turned 40 or 50 years, during 1990 to 2000 (ie, born 1940–50). Of the 56 977 eligible individuals, 34 269 (60.1%) completed the health examination.

A detailed overview of the selection procedure for the present study is presented in online supplemental figure 1. Reasons for exclusion were as follows: people who had moved from the county, missing records of one or more components related to the metabolic syndrome, incomplete date of health examination or death, previous record of coronary heart disease diagnosis or stroke diagnosis, for complete information on International Classification of Diseases (ICD) codes, see supplements, table 1. Finally, individuals who were deregistered from the Swedish Tax Agency Population Registration, who had deviant age or missing records for one or more of the covariates, were excluded. In total, 8371 individuals were lost to follow-up, leaving 25 898 individuals eligible for a matching procedure. Individuals that met a modified criteria for MetS were identified, see paragraph ‘Definition of the metabolic syndrome’. Participants identified with MetS were matched to two controls regarding sex, age and date of health examination. We matched participants who, by the time of the health examination, were 39, 40 or 41 years and 49, 50 or 51 years old respectively. Time of health examination was dichotomised to two groups, performed 1989–94 or 1995–2000, with a vast majority of the matched controls having had their health examination the same year as the individual with MetS to whom they were matched.

**Table 1:** Stratified crude HR and 95% CI for all-cause mortality and cardiovascular events per 1-unit increase

	Women, 40 years old (n=1800)	Women, 50 years old (n=5304)	Men, 40 years old (n=2691)	Men, 50 years old (n=5457)	Matched cohort population,(n=15 252)
All-cause mortality					
Education	1.48^ns^ (0.90 to 2.44)	1.29** (1.09 to 1.51)	1.31^ns^ (0.89 to 1.93)	1.46*** (1.27 to 1.67)	1.38*** (1.25 to 1.53)
Single household	1.16^ns^ (0.55 to 2.47)	1.57*** (1.25 to 1.97)	1.64** (1.14 to 2.35)	1.89*** (1.58 to 2.27)	1.72*** (1.51 to 1.96)
Current smoker	1.93*** (1.30 to 2.84)	2.34*** (1.99 to 2.75)	2.27*** (1.70 to 3.05)	2.22*** (1.93 to 2.57)	2.25*** (2.04 to 2.48)
Body Mass Index (kg/m^2^)	1.07*** (1.03 to 1.11)	1.03*** (1.02 to 1.05)	1.05** (1.01 to 1.08)	1.04*** (1.02 to 1.06)	1.04*** (1.03 to 1.05)
Hip circumference (10 cm)	1.30** (1.07 to 1.59)	1.14** (1.05 to 1.23)	1.13^ns^ (0.93 to 1.36)	1.11* (1.01 to 1.23)	1.14*** (1.08 to 1.21)
Mean arterial pressure (10 mm Hg)	1.12^ns^ (0.95 to 1.33)	1.16*** (1.09 to 1.23)	1.23** (1.08 to 1.40)	1.24*** (1.18 to 1.31)	1.20*** (1.16 to 1.25)
Metabolic syndrome	1.45* (1.04 to 2.02)	1.57*** (1.39 to 1.78)	1.54*** (1.22 to 1.95)	1.38*** (1.23 to 1.54)	1.47*** (1.36 to 1.58
Factor 1 blood pressure	1.17^ns^ (0.96 to 1.42)	1.22*** (1.14 to 1.31)	1.22** (1.07 to 1.40)	1.29*** (1.21 to 1.38)	1.25*** (1.20 to 1.31)
Factor 2 cholesterol	1.18* (1.02 to 1.37)	1.16*** (1.10 to 1.23)	1.27*** (1.14 to 1.41)	1.10*** (1.05 to 1.16)	1.14*** (1.11 to 1.18)
Factor 3 blood glucose	1.30** (1.09 to 1.56)	1.24*** (1.15 to 1.33)	1.11^ns^ (0.98 to 1.25)	1.16*** (1.09 to 1.23)	1.19*** (1.14 to 1.24)
Cardiovascular event					
Education	1.56* (1.02 to 2.38)	1.27** (1.08 to 1.48)	1.23^ns^ (0.92 to 1.65)	1.13^ns^ (0.99 to 1.28)	1.20*** (1.10 to 1.32)
Single household	1.30^ns^ (0.78 to 2.16)	1.07^ns^ (0.90 to 1.27)	0.87^ns^ (0.68 to 1.11)	1.12^ns^ (0.99 to 1.28)	1.11* (1.01 to 1.22)
Current smoker	1.66** (1.18 to 2.34)	1.56*** (1.34 to 1.81)	1.47*** (1.18 to 1.85)	1.44*** (1.26 to 1.64)	1.50*** (1.37 to 1.63)
Body Mass Index (kg/m^2^)	1.10*** (1.06 to 1.14)	1.06*** (1.04 to 1.08)	1.05*** (1.02 to 1.08)	1.05*** (1.03 to 1.06)	1.06*** (1.05 to 1.07)
Hip circumference (10 cm)	1.52*** (1.27 to 1.82)	1.28*** (1.18 to 1.38)	1.15^ns^ (0.99 to 1.33)	1.16** (1.06 to 1.27)	1.24*** (1.18 to 1.31)
Mean arterial pressure (10 mm Hg)	1.50*** (1.30 to 1.73)	1.23*** (1.16 to 1.30)	1.28*** (1.16 to 1.41)	1.23*** (1.17 to 1.29)	1.25*** (1.21 to 1.29)
Metabolic syndrome	2.49*** (1.89 to 3.30)	1.71*** (1.51 to 1.93)	1.75*** (1.46 to 2.09)	1.39*** (1.26 to 1.54)	1.59*** (1.49 to 1.70)
Factor 1 blood pressure	1.57*** (1.34 to 1.84)	1.31*** (1.22 to 1.41)	1.25*** (1.12 to 1.38)	1.27*** (1.20 to 1.34)	1.30*** (1.25 to 1.35)
Factor 2 cholesterol	1.50*** (1.28 to 1.76)	1.25*** (1.17 to 1.34)	1.31*** (1.19 to 1.45)	1.22*** (1.15 to 1.29)	1.26*** (1.21 to 1.31)
Factor 3 blood glucose	1.24** (1.06 to 1.45)	1.19*** (1.12 to 1.27)	1.09^ns^(0.98 to 1.21)	1.10** (1.04 to 1.16)	1.14*** (1.09 to 1.18)

Crude hazard ratioHR_ co variates and factors 1_–3.

Each stratum contains one MetS individual and two individuals matched on age, sex, and time of inclusion.

Data presented with crude hazard ratioHR and (95% CI CI). ns, non-significant, *p, **p, ***p.

Education: post-elementary school= 0, elementary school= 1. Current smoker: never smoker=0, former smoker or current smoker=1. Single household: 0=more than one person in household, 1= single household.

*p<0.05.

**p<0.01.

***p<0.001.

nsnon-significant

Follow-up started at the date of the index examination of an individual and ended in June 2022.

According to Swedish ethical standards in 1988–89, the invitation to participate in the health screening included written information on that data, including test results, should be filed and used for future scientific studies. The information was repeated verbally by the attending nurse to those who chose to participate in the health screening. An application was filed to the Regional Board of Ethics, Uppsala in 2007 to ensure that future studies should be in line with more recent ethical standards. The Uppsala Regional Ethical Review Board approved the study by waiving informed consent (Dnr 2007/165, 2020–0930).

### Data collection

The health screening involved a clinical examination by a primary healthcare nurse. The screening included measurements of height, weight, office systolic and diastolic blood pressure, total cholesterol, blood glucose, waist and hip circumference. Blood pressure was measured with a manual sphygmomanometer after 15 min of rest in a sitting position*.* ‘Mean arterial pressure’ (MAP) was used to further analyse the association between high SPB and DBP. The definition of mean arterial pressure is the average arterial pressure throughout one cardiac cycle, systole and diastole. Mean arterial blood pressure was calculated by using the formula SBP+2*(DBP)/3.^10^ Blood cholesterol and blood glucose were measured after 2 hours of refraining from eating and were analysed using a factory-calibrated photometer. Body Mass Index was calculated as the body mass divided by the square of the body height (kg/m^2^). At this visit, the participant also handed in a self-administered questionnaire that included, among others, questions about lifestyle habits, previous history of CVD and diabetes mellitus and socioeconomic factors. Education was self-reported as number of years post-elementary school. In general, this implies 7 years in elementary school for participants born in the 1940s and 9 years for participants born in the 1950s due to a change in the Swedish school system. Occupation was self-reported in free text. Due to missing records of education in 285 of the participants with MetS, data was estimated from occupation. This was performed in three steps—first, all participants with missing data on education were identified. Second, all authors of this paper assessed the likely level of education in relation to the participants’ occupation. Third, the first author and the data manager compiled this information and made an overall assessment of the participants’ level of education. Discrepancies were solved in consensus after discussion with all the authors.

### Definition of the metabolic syndrome

Individuals that met three or more of the following risk factors were classified with MetS: waist circumference: ≥102 cm (men) and ≥88 cm (women), total cholesterol: ≥6.1 mmol/L, blood pressure: ≥130 and/ or ≥85 mm Hg (or previous diagnosis of hypertension) and plasma glucose: ≥5.6 mmol/L (or previous diagnosis of type 2 diabetes). Since the data collection did not allow a definition including high-density lipoprotein (HDL) and triglycerides, total cholesterol was used as a marker for dyslipidaemia.

### Coronary heart disease and stroke diagnosis and CVD-related death

Data from the National Board of Health and Welfare (NBHW) were used for the years 1963–2007. In addition, a local register, the DUVA (Datalager för Uppföljning och VerksamhetsAnalys) 2008–22, was our main diagnoses register since it comprises data from both primary healthcare and hospitals in the county of Västmanland. Participants in the study were considered to have a diagnosis of CVD if they had a record of any of the ICD-10-SE codes related to ischaemic heart disease and/or cerebrovascular disease both shown in online supplemental table 1. Since ICD-10-SE was implemented in 1997, a conversion of the diagnoses to ICD-9-SE and ICD-8-SE was performed to be used for the earlier registers. Conversions tables supplied by the NBHW were used for this procedure and all ICD-9-SE and ICD-8-SE are shown in online supplemental table 1. Finally, the national register for death causes was used. Both primary and secondary diagnoses were searched for in all registers.

### Patient and public involvement

There was no patient and public involvement in the study.

### Statistical analysis

All continuous variables are presented as median (Q1, Q3). Categorical variables are presented as frequency and percents. Smoking was dichotomised to never smoker vs ex-smoker/current smoker. Members in household were dichotomised to living alone vs more than one person in the household. And finally, education was dichotomised to lower education (elementary school +0.9 years) vs higher education (>0.9 years post-elementary school).

Associations between the components of MetS (waist circumference, total cholesterol, blood glucose, systolic and diastolic blood pressure) were assessed using Spearman rank correlation, which measures the monotone relation between two variables. The factor analysis included the variables used to define the metabolic syndrome. Blood glucose was skewed and therefore, log-transformed prior to the factor analysis. Principal component analysis within each age and sex strata was used to identify the initial set of uncorrelated factors. The scree test was used to determine the number of factors to retain. It uses the graph of descending eigenvalues, λi vs i, and selects as the number of factors to retain the value of i corresponding to an ‘elbow’ in the curve; this is considered to be the point at which large eigenvalues cease and small eigenvalues begin. To simplify the interpretation of the selected factors, the varimax (orthogonal) rotation was used. This has as its rationale the provision of uncorrelated factors with a few large loadings and as many near-zero loadings as possible. The cut-off for loadings was set at 0.35.

Crude and adjusted prospective associations of MAP, MetS and the factors retained from the factor analysis and first cardiovascular (CV) event and all-cause mortality were analysed for the total study population and by sex and age group separately, using stratified Cox proportional hazards regression models, where each stratum contains one MetS individual and two controls individually matched on age, sex and health examination.^11^ Adjusting variables were smoking, education, single household, BMI and hip circumference.

The proportional hazards assumption was assessed by visual inspection of the log[-log(cumulative survival)] for each categorical variable and by visual inspection of the Schoenfeld residuals vs time for continuous variables. Cumulative survival was estimated by the Kaplan-Meier estimator.

Kaplan-Meier survival analysis was used to assess the time to first CV event and all-cause mortality for individuals with or without MetS and for individuals within the highest quartile of MAP compared with the three lower quartiles.

A two-sided p value <0.05 was regarded as statistically significant. IBM SPSS Statistics, V.28 was used for all analyses.

## Results

A total of 5084 individuals that met the criteria for MetS were identified. They were individually matched to two controls without MetS (n=10 168) with regard to age, sex and date of health examination. Of the total study population (n=15 252), 29% were turning 40 years and 47% were female (online supplemental table 2).

The mean (Q1, Q3) follow-up time for mortality was 26.8 years (24.1, 29.8) and 27.1 years (24.7, 30.41) and corresponding to 136 251 and 275 553 person-years at risk in the MetS and the control group respectively. There were 1645 (32.4%) CV events in the MetS group and 2321 (22.8%) CV events for the controls corresponding to 12 CV events in the MetS group and 8 CV events in the controls per 1000 person-years at risk. During follow-up, 1317 (25.9%) MetS and 1904 (18.7%) control subjects died, implying 10 deaths in the MetS group and 7 deaths in the controls per 1000 person-years at risk. The proportion of individuals with MetS was higher for both outcomes in both age groups and both sexes. For further information see online supplemental table 3.

The results of the Cox regression analysis of covariates demonstrated that when MetS was present, the crude HR was 1.59*** (95% CI 1.49 to 1.70) for CV event and 1.47*** (95% CI 1.36 to 1.58) for all-cause mortality (table 1).

A factor analysis was performed to explore the effects from the individual components related to MetS. This included a Spearman rank correlation analysis of the metabolic syndrome components stratified by sex and age. Three dominating factors were identified, which explained 80% of the total variation among women and 78% among men. After varimax rotation, factor 1, the blood pressure factor consisted of systolic and diastolic blood pressure and waist circumference for both sexes and both age groups. Factor 1 explained between 38.6% and 42.6% of the total variance for the different sex and age groups. For the younger age group, factor 2, the cholesterol factor, consisted of cholesterol and waist circumference for both sexes. Factor 3, the blood glucose factor, consisted of log blood glucose for both sexes for the younger age group. For the older age group, factors 2 and 3 were opposite than for 40-year-olds that is, factor 2=blood glucose and factor 3=cholesterol. For further information, see online supplemental table 4.

The Cox regression analysis showed that factor 1 (blood pressure) increased the risk for CV events with 30% (HR 1.30*** 95% CI 1.25 to 1.35) and for all-cause mortality with 25% (HR 1.25*** 95% CI 1.20 to 1.31) for participants with MetS. For women at the age of 40, factor 1 (blood pressure) increased the risk for CV events with 57% (HR 1.57*** 95% CI 1.34 to 1.84), more than doubled compared with men at the same age (HR 1.25*** 95% CI 1.12 to 1.38) (table 1).

The adjusted HRs for first CV event and death were 1.39*** (95% CI 1.28 to 1.50) and 1.27*** (95% CI 1.16 to 1.40) when MetS was present and 1.19*** (95% CI 1.15 to 1.23) and 1.16*** (95% CI 1.11 to 1.21) respectively for the matched cohort population for each 10 mm Hg increased MAP. An increase in MAP for 40-year-old women was related to a higher risk for a CV event, HR 1.35*** (95% CI 1.15 to 1.57) compared with 50-year-old women and men from both age groups 1.15*** (95% CI 1.08 to 1.22), 1.26*** (95% CI 1.13 to 1.40) and 1.19*** (95% CI 1.13 to 1.26) respectively (table 2).

**Table 2:** Stratified adjusted HR and 95% CI for all-cause mortality and cardiovascular events per 1-unit increase. Each stratum contains one MetS individual and two individuals matched on age, sex and time of inclusion

	Women, 40 years (n=1800)	Women, 50 years (n=5304)	Men, 40 years (n=2691)	Men, 50 years (n=5457)	Matched cohort population(n=15 252)
All-cause mortality					
Mean arterial pressure	0.99^ns^ (0.81 to 1.20)	1.12*** (1.05 to 1.20)	1.18* (1.02 to 1.37)	1.20*** (1.13 to 1.28)	1.16*** (1.11 to 1.21)
Metabolic syndrome	0.90^ns^ (0.57 to 1.41)	1.40*** (1.19 to 1.63)	1.37* (1.02 to 1.84)	1.22** (1.07 to 1.39)	1.27*** (1.16 to 1.40)
Factor 1 blood pressure	1.00^ns^ (0.79 to 1.26)	1.22*** (1.12 to 1.34)	1.24** (1.05 to 1.46)	1.30*** (1.20 to 1.40)	1.24*** (1.17 to 1.31)
Factor 2 cholesterol	0.90^ns^ (0.71 to 1.14)	1.11* (1.03 to 1.20)	1.26** (1.07 to 1.49)	1.07^ns^ (1.00 to 1.15)	1.09*** (1.04 to 1.15)
Factor 3 blood glucose	1.18^ns^ (0.97 to 1.42)	1.23*** (1.14 to 1.32)	1.06^ns^ (0.93 to 1.21)	1.13*** (1.06 to 1.21)	1.16*** (1.11 to 1.21)
Cardiovascular event					
Mean arterial pressure	1.35*** (1.15 to 1.57)	1.15*** (1.08 to 1.22)	1.26*** (1.13 to 1.40)	1.19*** (1.13 to 1.26)	1.19*** (1.15 to 1.23)
Metabolic syndrome	1.84*** (1.29 to 2.63)	1.39*** (1.20 to 1.61)	1.67*** (1.35 to 2.07)	1.29*** (1.15 to 1.44)	1.39*** (1.28 to 1.50)
Factor 1 blood pressure	1.46*** (1.21 to 1.77)	1.24*** (1.14 to 1.35)	1.28*** (1.13 to 1.44)	1.27*** (1.18 to 1.36)	1.26*** (1.20 to 1.32)
Factor 2 cholesterol	1.30** (1.08 to 1.58)	1.17*** (1.08 to 1.26)	1.30*** (1.15 to 1.46)	1.19*** (1.12 to 1.27)	1.20*** (1.15 to 1.25)
Factor 3 blood glucose	1.15^ns^ (0.96 to 1.37)	1.14*** (1.06 to 1.23)	1.06^ns^ (0.95 to 1.18)	1.08* (1.02 to 1.15)	1.10*** (1.06 to 1.15)

Adjusted hazard ratioHR MAP, MetS and Ffactors 1_–3.

Data presented with adjusted HRs for 1-unit increase. Adjusted for: smoking, education, single household, body mass index and hip circumference. * p, ** p, *** p=, -significant.

*p<0.05.

**p<0.01.

***p= <0.001.

nsnon-significant

**Figure 1:**
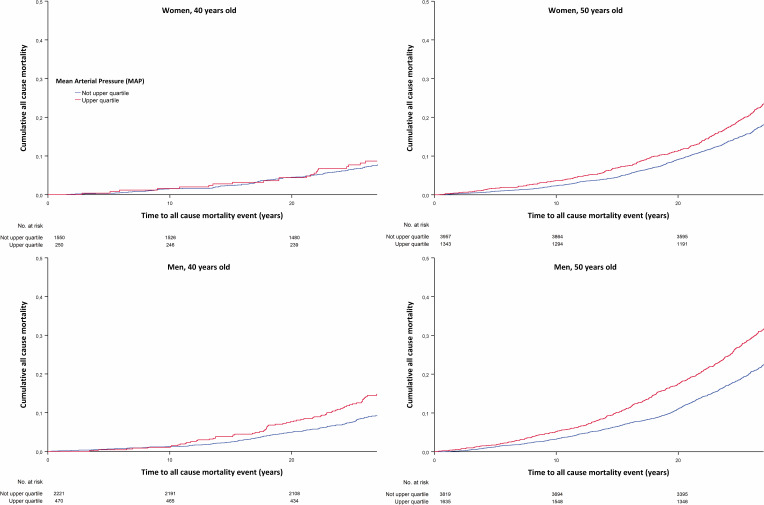
Kaplan-Meier analysis mean arterial pressure and all-cause mortality.

To further elaborate how blood pressure affects all-cause mortality and CVD morbidity, a Kaplan-Meier analysis was performed to examine the impact of MAP on CV morbidity and all-cause mortality. Mean time for first CV event and/or death was 2.6 years and 1.5 years shorter respectively for participants within the highest quartile compared with participants with lower mean arterial blood pressure for the total study population (figures1  2).

**Figure 2:**
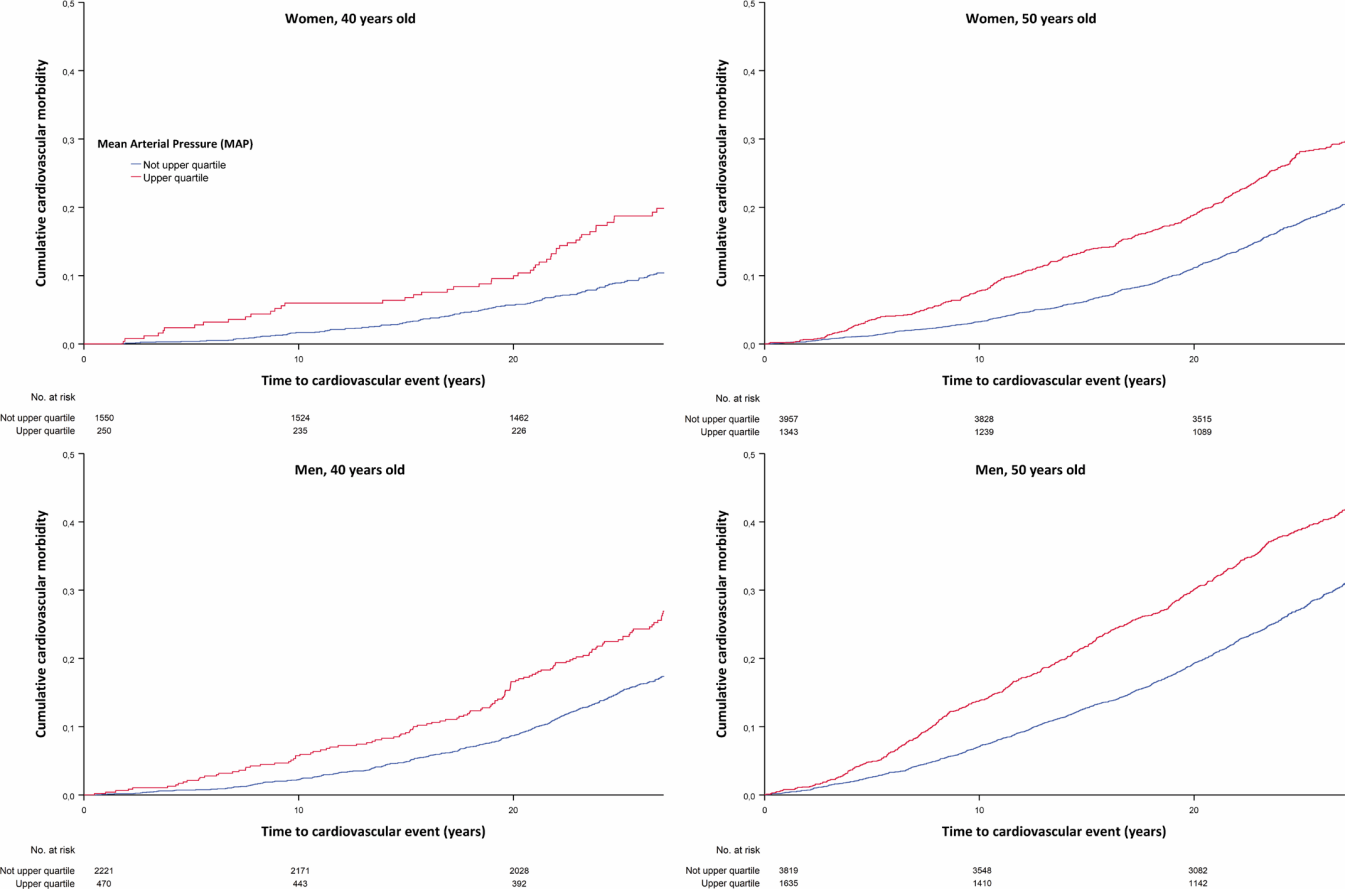
Kaplan-Meier analysis mean arterial pressure and CV event. CV, cardiovascular.

## Discussion

The main finding of this population-based matched cohort study was that middle-aged individuals with asymptomatic MetS have increased risk for CVD morbidity with 39% and all-cause mortality with 27% after 27 years follow-up, with a 2.3-year difference to first CV event. Moreover, the results demonstrated that blood pressure was identified as the MetS component with the strongest association to CV events, and also, that individuals within the highest quartile of MAP in midlife had significantly shorter time to first CV event compared with the lower three quartiles.

It is well-established that the metabolic syndrome predicts CVD and DMT2, although there are different results whether the risk associated with MetS exceeds its individual components.^12^,^14^ To increase the understanding of how the different components included in MetS are associated with all-cause mortality and CV events, we performed immersed analyses that included a factor analysis and Cox regression analyses on mean arterial blood pressure and the retained factors. The blood pressure component has previously been demonstrated to be the most prominent component in MetS with regard to both CVD-related morbidity and all-cause mortality.^15^ A pooled cohort study including over 1 500 000 participants, performed by the Global Cardiovascular risk consortium, studied the association between five risk factors (BMI, systolic blood pressure, non-high-density lipoprotein cholesterol, current smoking and diabetes) and the 10-year incidence of CVD. These five modifiable risk factors accounted for an aggregate global population-attributable fraction of the 10-year incidence of cardiovascular disease of 57.2% (95% CI, 52.4 to 62.1) among women and 52.6% (95% CI, 49.0 to 56.1) among men. Out of the five risk factors, high systolic blood pressure was considered to be the leading risk factor for CVD and could therefore, offer the greatest potential for the prevention of CVD, when controlled.^16^

Hypertension is a major cause of premature death worldwide. Half of the population with elevated blood pressure remain undiagnosed according to a study from the non-commutable disease risk factor collaboration. According to the same study, half of those who are diagnosed with hypertension are offered blood pressure-lowering treatment and less than half of those treated had achieved therapeutic goal. Hence, global control rates regarding blood pressure are low, 23% for women and 18% for men with hypertension.^17^ Most hypertension and cardiovascular risk prevention guidelines advocate a combination of lifestyle change and pharmacotherapy.^18^,^20^ However, adherence to risk factor control is poor according to studies from both European Action on Secondary and Primary Prevention by Intervention to Reduce Events (EUROASPIRE) V^21^ and Sweden.^22^ The study from EUROASPIRE revealed that large proportions of people at high risk for CVD have inadequate control of their blood pressure and unhealthy lifestyle habits at the same time.^21^

The blood pressure factor in our study consisted of both systolic, diastolic blood pressure and waist circumference. Central obesity is a chronic metabolic disorder and part of the metabolic syndrome. It has been suggested that BMI and waist circumference are involved in the aetiology of hypertension and previous studies have demonstrated an increased risk for hypertension when waist circumference is elevated.^23 24^ Even if the causation is not explained by the factor analysis, our results are in line with previous studies and underline the importance to include measurement of waist circumference in screening programme for CV risk factors.

Both the metabolic syndrome and elevated blood pressure are conditions that will remain asymptomatic and therefore, undiagnosed for long periods of time. As a consequence, many patients do not have any symptoms of CVD before a serious first event, such as myocardial infarction or stroke. In our study 40-year-old women with high blood pressure presented the highest risk from CV events later in life, an increased risk with 40% compared with men at the same age and to 50-year-old participants, who were at about 20% higher risk for CV events.

Identifying asymptomatic individuals for preventive treatment may reduce risk for future CVD morbidity and mortality. Public health screenings, directed to different age groups, are one example of how preventive efforts can be performed to identify cardiovascular risk factors. Previous studies have examined the effects on Swedish health screening programmes for cardiovascular risk factors and demonstrated both decreased mortality rates and cardiovascular events.^25^,^28^

However, there has been a long-running discussion in the scientific community about the possibility of using prevention programmes to reduce the CVD burden in the population. The conclusion based on, for example, Cochrane evaluations of multiple RCTs, is that there is limited evidence for CVD prevention efforts to be effective. On the other hand, the same Cochrane review stated that: ‘Overall, systematic risk assessment appears to result in lower total cholesterol levels, lower systolic blood pressure and lower diastolic blood pressure’.^29^ In a review by Liss *et al*, the conclusion was that general health checks were not associated with reduced mortality or cardiovascular events but were associated with increased chronic disease recognition and treatment, risk factor control, preventive service uptake and improved patient-reported outcomes.^30^ This was further underlined in a study performed on the behalf of the World Stroke Organisation. One of their conclusions was to implement both primary and secondary prevention strategies with special emphasis on early detection and control of hypertension to reduce the global burden of stroke.^31^ The Swedish model of health screening programmes combined with community-based interventions has been evaluated several times. One large scale, still ongoing screening programme in northern Sweden, has been evaluated in terms of cost-effectiveness by Lindholm *et al* in 2018. Their conclusion was that the community-based intervention programme from the county of Västerbotten was ‘-extremely cost-effective in relation to the Swedish threshold value per QALY gained’.^32^

Screening for CVD risk factors had its glory days in the 1990s, since then, many voices have questioned this due to costs and displacement effects.^33^ With our longitudinal population-based matched cohort study, we found that about 20% of middle-aged men and women had asymptomatic MetS. The time to first cardiovascular events was about 2.3 years shorter for individuals with MetS, which gives us ‘a windows of opportunity’ to intervene and prevent serious cardiovascular events and premature death.

### Strengths and limitations

Effectiveness assessment based on impact on morbidity and mortality requires a long-term follow-up and a sufficiently large scale to be able to measure impact on rare events such as mortality in middle age. This is the case for WICTORY, the underlying health screening programme for our study, which was both large scale and presents a follow-up time that exceeds 30 years for some of the participants. This long follow-up time and study design as a prospective matched cohort has given an opportunity to analyse the long-term effects of CV risk factors and blood pressure in more detail.

Although this population-based study with large number of participants and long follow-up time, along with a robust statistical analysis, has several strengths, there are also limitations.

The screening for CVD risk factors may have induced pharmacological treatment, especially, for the participants with the highest blood pressure. As the data does not provide this information, it is possible that pharmacological treatment may have had an impact on the results. In addition, the situation regarding risk factors, drug treatment and time-trends for lifestyle habits may have shifted from the beginning of 1990 to the end of 1999. However, we aimed to address the time-dependent variation by stratification where each stratum contained one MetS individual and two individuals matched on age, sex and time of inclusion.

Second total cholesterol comprises both HDL, low-density lipoprotein (LDL) and triglycerides. This makes it harder to evaluate to what extent cholesterol affected the outcome. As HDL and LDL have opposite effects on the atherosclerotic process, this might have influenced the impact of total cholesterol. Blood glucose was measured after 2 hours of refraining from eating. This might also influence our results as this makes it difficult to interpret whether this was a true fasting blood glucose or not.

Third, information on physical activity was self-reported by a non-validated question included in the questionnaire. Our intention was to include physical activity as a covariate in the adjusted Cox regression regarding MetS, MAP and the retained factors 1–3. However, as the preliminary analyses demonstrated diverse and insignificant results, this was not possible, and therefore, omitted from the final analyses.

Fourth, generalisability is limited due to a homogenous population, mainly Caucasian men and women, even if the population in the county of Västmanland is a good representation of the Swedish population. The results may therefore be generalisable to other western European countries and Caucasian population in North America.

## Conclusion

The risk of a CV event and premature death later in life is significantly increased when asymptomatic MetS is present in midlife. Blood pressure had the strongest association to both CV events and all-cause mortality compared with the other components included in MetS. The results add to previous knowledge that early detection, that is, with population-based screening programmes for metabolic risk factors is of importance to detect these silent but harmful risk factors and illuminates the importance of reaching treatment goals for blood pressure to reduce the burden of CVD and all-cause mortality.

## supplementary material

10.1136/bmjopen-2023-081444 online supplemental table 1

10.1136/bmjopen-2023-081444 online supplemental table 2

10.1136/bmjopen-2023-081444 online supplemental table 3

10.1136/bmjopen-2023-081444 online supplemental table 4

10.1136/bmjopen-2023-081444 online supplemental figure 1

10.1136/bmjopen-2023-081444 Uncited online supplemental file 1

## Data Availability

Data are available upon reasonable request.
