# Supplementary material for: Occurrence of metabolic syndrome in midlife in relation to cardiovascular morbidity and all-cause mortality—lessons from a population-based matched cohort study with 27 years follow-up
Source: BMJ Open. 2024 Sep 16;14(9):e081444. doi: 10.1136/bmjopen-2023-081444 (PMC11409331; doi:10.1136/bmjopen-2023-081444)
Supplement: online supplemental table 1 [file bmjopen-14-9-s001.pdf]

Supplemental Table 1. Ischaemic heart disease and stroke diagnoses

ICD 10-SE	ICD 9-SE	ICD 8-SE
I20 Angina pectoris	413A, 413B, 413X<br>413.0, 413.1, 413.9	411-
I21 Acute myocardial infarction	410A, 410B, 410W, 410X<br>410.0, 410.1, 410.8, 410.9	410-,411-,426,429
I22 Subsequent ST elevation and non-ST elevation myocardial infarction	410.A, 410B, 410W, 410X<br>410.0, 410.1, 410.8, 410.9	410-,411-,426,429
I24 Other acute ischemic heart diseases	410A , 410B , 410W , 410X , 411A, 411B<br>410.0, 410.1, 410.8, 410.9, 411.0, 411.1	410-,411-,412-,426,429
I25 Chronic ischemic heart disease	412X, 414A, 414B, 414W, 414X<br>412.9, 414.0, 414.1, 414.8, 414.9	410-,412-,414-,429,4589
I61 Nontraumatic intracerebral haemorrhage	431X<br>431.9	431-
I62 Other and unspecified nontraumatic intracranial haemorrhage	432A, 432B, 432X<br>432.0, 432.1, 432.9	431
I63 Cerebral infarction	434A, 434.B, 434.X, 437A<br>434.0, 434.1, 434.9, 437.0	433-,434-,437-,4389
I64 Stroke, not specified as haemorrhage or infarction	436X<br>436.9	4369
I65 Occlusion and stenosis of precerebral arteries, not resulting in cerebral infarction	433A, 433B, 433C, 433D, 433W, 433X<br>433.0, 433.1, 433.2, 433.3, 433.8, 433.9	432-
I66 Occlusion and stenosis of cerebral arteries, not resulting in cerebral infarction	434A, 434B, 434X, 437A, 437B<br>434.0, 434.1, 434.8, 437.0, 437.1	433-,434-,437-,4389
G45 Transient cerebral ischemic attacks and related syndromes	435X<br>435.9	433
