# Supplementary material for: Occurrence of metabolic syndrome in midlife in relation to cardiovascular morbidity and all-cause mortality—lessons from a population-based matched cohort study with 27 years follow-up
Source: BMJ Open. 2024 Sep 16;14(9):e081444. doi: 10.1136/bmjopen-2023-081444 (PMC11409331; doi:10.1136/bmjopen-2023-081444)
Supplement: online supplemental table 2 [file bmjopen-14-9-s002.pdf]

Supplemental Table 2. Baseline characteristics of study participants.

	Women, 40 years old (n=1 800)		***Women, 50 years old (n=5 304)***		***Men, 40 years old (n=2 691)***		***Men, 50 years old (n=5 457)***		***Matched cohort population (n=15 252)***	
	MetS (n=1200)	*Non-MetS (n=600)*	*MetS (n=1768)*	*Non-MetS (n=3536)*	*MetS (n=897)*	*Non-MetS (n=1794)*	*MetS (n=1819)*	*Non-MetS (n=3638)*	*MetS (n=5084)*	*Non-MetS (n=10168)*
**Systolic blood pressure, mmHg**	135 (130, 141.5)	120 (110, 130)	140 (130, 152)	126.5 (120, 140)	136 (130, 145)	125 (120, 135)	140 (130, 150)	130 (120, 140)	140 (130, 150)	125 (120, 140)
**Diastolic blood pressure, mm Hg**	85 (80, 90)	80 (70, 85)	90 (82, 95)	80 (75, 88)	89 (85, 95)	80 (75, 85)	90 (85, 95)	84 (80, 90)	90 (85, 95)	80 (75, 90)
**Total Cholesterol, mmol/L**	6.2 (5.4, 6.8)	5 (4.4, 5.6)	6.5 (6.0, 7.1)	5.5 (4.9, 6.1)	6.5 (6.1, 7.0)	5.3 (4.6, 6.0)	6.6 (6.1, 7.2)	5.6 (5.0, 6.3)	6.5 (6.1, 7.1)	5.4 (4.8, 6.1)
**Mean arterial blood pressure (MAP), mmHg**	10.3 (9.7, 10.7)	9.2 (8.7, 10.0)	10.6 (10.0, 11.3)	9.7 (9.0, 10.4)	10.3 (9.8, 11.0)	9.5 (9.0, 10.1)	10.7 (10.0, 11.3)	9.8 (9.3, 10.7)	10.5 (10.0, 11.2)	9.7 (9.0, 10.3)
**Plasma glucose, mmol/L**	5.9 (5.6, 6.5)	5.3 (4.8, 5.7)	5.9 (5.6, 6.4)	5.3 (4.9, 5.7)	5.9 (5.6, 6.6)	5.3 (4.9, 5.8)	6.0 (5.6, 6.7)	5.3 (4.9, 5.9)	5.9 (5.6, 6.6)	5.3 (4.9, 5.8)
**Waist circumference, cm**	92 (87, 100)	76 (72, 83)	92 (86, 99)	78 (73, 84)	103 (94, 108)	90 (85, 96)	102 (94, 107)	92 (87, 98)	97 (90, 105)	86 (78, 93)
**Hip circumference, cm**	109 (103, 116)	100 (96, 106)	109 (103, 115)	101 (97, 106)	107 (103, 111)	102 (99, 106)	107 (102, 111)	102.5 (99, 106)	108 (103, 113)	102 (98, 106)
**Body Mass Index, kg/m^2**	28.7 (26.4, 32.4)	23.4 (21.9, 25.9)	28.8 (26.1, 32.0)	24.2 (22.3, 26.4)	28.6 (25.8, 31.0)	24.9 (23.1, 26.6)	28.3 (26.0, 30.7)	25.4 (23.7, 27.4)	28.6 (26.0, 31.3)	24.7 (22.8, 26.8)
**Current smoker, n (%)**	231 (38.5)	387 (32.3)	572 (32.4)	1054 (29.8)	270 (30.1)	462 (25.8)	521 (28.6)	982 (27)	1594 (31.4)	2885 (28.4)
**Living alone, n (%)**	43 (7.2)	55 (4.6)	186 (10.5)	409 (11.6)	142 (15.8)	244 (13.6)	228 (12.5)	445 (12.2)	599 (11.8)	1153 (11.3)
**Lower education, n (%)**	141 (23.5)	141 (11.8)	649 (36.7)	850 (24.0)	177 (19.7)	209 (11.6)	625 (34.4)	893 (24.5)	1592 (31.3)	2093 (20.6)

Variables are presented with median (Q1, Q3) or frequency and (percentage). MetS= Individuals that met three or more of the following risk factors were classified with metabolic syndrome (MetS): waist circumference: ≥102 cm (men) and ≥88 cm (women), total cholesterol: ≥6.1 mmol/ l, blood pressure: ≥130 and/ or ≥85 mm Hg (or previous diagnosis of hypertension), plasma glucose: ≥5.6 mmol/ l (or previous diagnosis of type 2 diabetes).
