# Supplementary material for: Occurrence of metabolic syndrome in midlife in relation to cardiovascular morbidity and all-cause mortality—lessons from a population-based matched cohort study with 27 years follow-up
Source: BMJ Open. 2024 Sep 16;14(9):e081444. doi: 10.1136/bmjopen-2023-081444 (PMC11409331; doi:10.1136/bmjopen-2023-081444)
Supplement: online supplemental table 3 [file bmjopen-14-9-s003.pdf]

**Supplemental Table 3. Outcome cardiovascular events and all-cause mortality**

| Outcome | *Women, 40 years old (n=1800)* | | *Women, 50 years old (n=5304)* | | *Men, 40 years old (n=2691)* | | *Men, 50 years old (n=5457)* | | *Matched cohort population (n= 15 252)* | |
|---|---|---|---|---|---|---|---|---|---|---|
| | *Non MetS* | *MetS* | *Non MetS* | *MetS* | *Non MetS* | *MetS* | *Non MetS* | *MetS* | *Non MetS* | *MetS* |
| **All-cause mortality, n (%)** | 94 (7.8) | 69 (11.5) | 672 (19.0) | 504 (28.5) | 182 (10.1) | 135 (15.1) | 956 (26.3) | 609 (33.5) | 1904 (18.7) | 1317 (25.9) |
| **CVD* (stroke and/ or CHD), n (%)** | 114 (9.5) | 122 (20.3) | 705 (19.9) | 535 (30.3) | 316 (17.6) | 242 (27.0) | 1186 (32.6) | 746 (41.0) | 2321 (22.8) | 1645 (32.4) |

Data presented with frequency and (percentage). * CVD diagnosis: ICD I20-22, ICD I24,25, I61-66, G45
