# Supplementary material for: Occurrence of metabolic syndrome in midlife in relation to cardiovascular morbidity and all-cause mortality—lessons from a population-based matched cohort study with 27 years follow-up
Source: BMJ Open. 2024 Sep 16;14(9):e081444. doi: 10.1136/bmjopen-2023-081444 (PMC11409331; doi:10.1136/bmjopen-2023-081444)
Supplement: online supplemental table 4 [file bmjopen-14-9-s004.pdf]

Supplemental Table 4. Factor Loadings

Individual component	*FACTOR (40 years old)*					
	Women			*Men*		
	(1) Blood pressure	*(2) Cholesterol*	*(3) Blood glucose*	*(1) Blood pressure*	*(2) Cholesterol*	*(3) Blood glucose*
**Waist**	**.472**[a]	**.403**	.336	**.478**	**.436**	.111
**Systolic bp**	**.890**	.089	.076	**.869**	.043	.114
**Diastolic bp**	**.901**	.094	-.001	**.893**	.109	-.060
**Total Cholesterol**	.093	**.960**	-.016	.055	**.946**	.003
**Log blood glucose**	.043	.005	**.970**	.052	.037	**.993**
	FACTOR (50 years old)					
	Women			*Men*		
	(1) Blood pressure	*(2) Blood glucose*	*(3) Cholesterol*	*(1) Blood pressure*	*(2) Blood glucose*	*(3) Cholesterol*
**Waist**	**.527**[a]	.310	.289	**.491**	.250	.234
**Systolic bp**	**.900**	.035	.019	**.894**	.040	.005
**Diastolic bp**	**.908**	-.051	.038	**.909**	-.085	.033
**Total Cholesterol**	.070	-.008	**.975**	.074	-.026	**.978**
**Log blood glucose**	.023	**.970**	-.012	.028	**.974**	-.028

[a]Loadings of the individual components included in the respective factor in bold (cut off= 0.35)
