# Supplementary figures and images for: Occurrence of metabolic syndrome in midlife in relation to cardiovascular morbidity and all-cause mortality—lessons from a population-based matched cohort study with 27 years follow-up
Source: BMJ Open. 2024 Sep 16;14(9):e081444. doi: 10.1136/bmjopen-2023-081444 (PMC11409331; doi:10.1136/bmjopen-2023-081444)

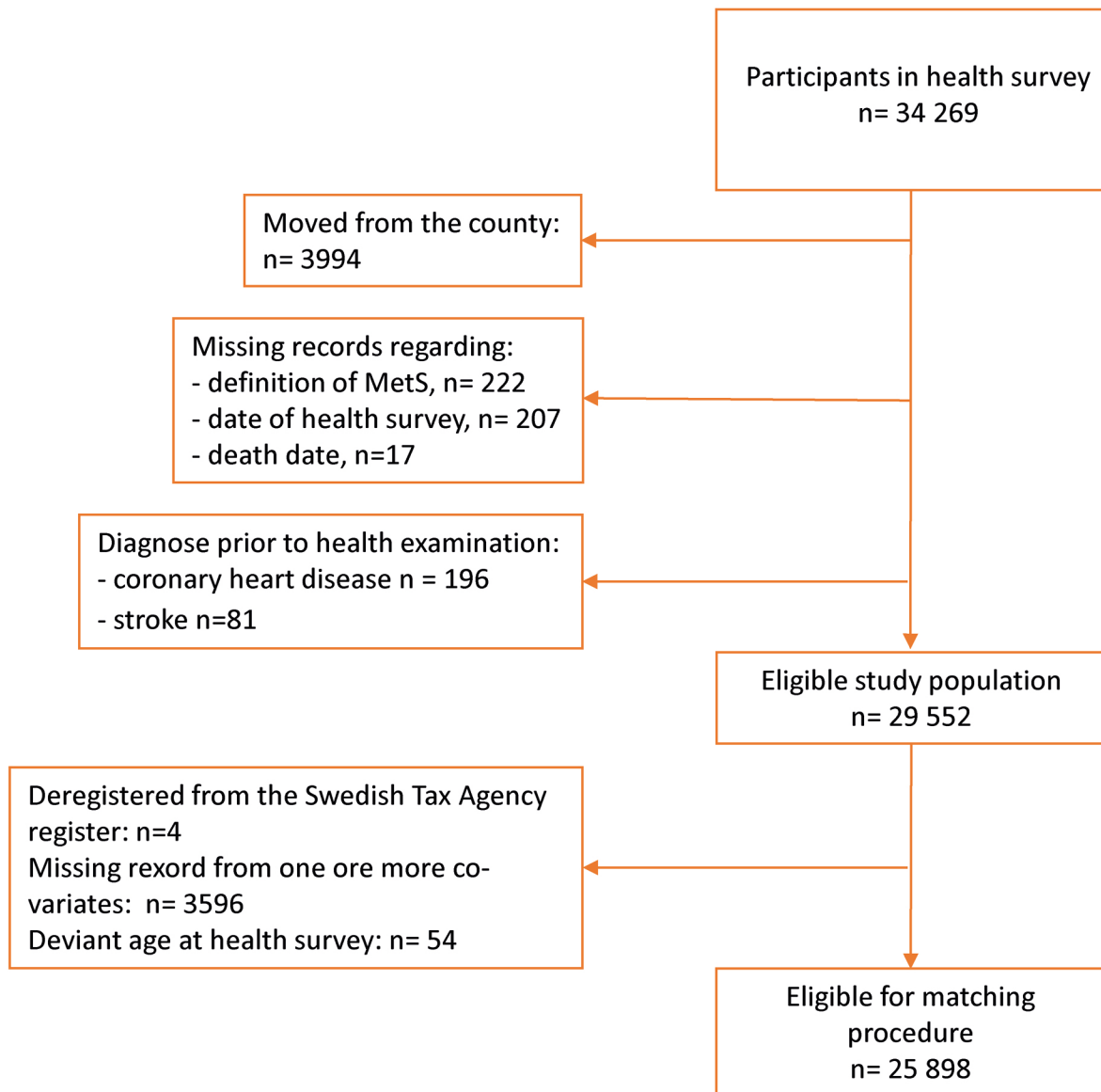

Supplement: online supplemental figure 1 [file bmjopen-14-9-s005.pdf]
