# Supplementary material for: Occurrence of metabolic syndrome in midlife in relation to cardiovascular morbidity and all-cause mortality—lessons from a population-based matched cohort study with 27 years follow-up
Source: BMJ Open. 2024 Sep 16;14(9):e081444. doi: 10.1136/bmjopen-2023-081444 (PMC11409331; doi:10.1136/bmjopen-2023-081444)
Supplement: Supplementary file 7 [file bmjopen-2023-081444.reviewer_comments.pdf]

PEER REVIEW HISTORY

BMJ Open publishes all reviews undertaken for accepted manuscripts. Reviewers are asked to complete a checklist review form and are provided with free text boxes to elaborate on their assessment. These free text comments are reproduced below.

ARTICLE DETAILS

**TITLE (PROVISIONAL)**	Occurrence of metabolic syndrome in midlife in relation to cardiovascular morbidity and all-cause mortality– lessons from a population-based matched cohort study with 27 years follow up.
**AUTHORS**	Lönnberg, Lena; Leppert, Jerzy; Ohrvik, John; Rehn, Mattias; Chabok, Abbas; Damberg, Mattias

VERSION 1 - REVIEW

**REVIEWER NAME**	Ishikawa, Shizukiyo
**REVIEWER AFFILIATION**	Jichi Medical University, Department of General Medicine
**REVIEWER CONFLICT OF INTEREST**	none
**DATE REVIEW RETURNED**	13-Dec-2023

**GENERAL COMMENTS**	This is the population-based study in which title the study design was case-control study with 27 years follow up. The study was with long interval in which all inhabitants turning 40 or 50 years between 1990-1999 were invited for screening. However, the reviewer has some concerns about the present study.<br><br>Is the present study case control study?<br>Case-control study is the design which examine to compare the base-line exposure based on between cases and controls with outcome. However, the present study defined cases or controls as participants Mets or without Mets. The reviewer thought the present study was a kind of cohort study, but not typical.<br><br>Why whole cohort was not used?<br>Case-cohort study was thought more convenient than whole cohort study because of the cost or some other difficult condition to obtain many covariates from whole cohort subjects. However, the authors used baseline data as total subjects of 34,269 (60.1%) people after screening by invitation and outcome of CV events and all-cause mortality from the index examination. The reviewer thought the whole cohort study was possible.<br><br>Prevalence of Mets at baseline was not proper for the present study, because controls were 1:2 matched to cases. Hazard ratios calculated in cohort study were analyzed using whole cohort because of proper prevalence of risk factors as study samples. However, using matched controls for case- cohort study, proper hazard ratios were not calculated.<br><br>The study subjects were only 40 and 50 years old men and women. Distribution of the ages of the subject were limited in 40 and 50 years; 10 years apart. The results should be separately shown in 40 and 50 years, not all data to calculate risk ratios.

	The reviewer was concerned that the situation around risk factors and outcome was changed or not for 10 years to obtain base line data.

**REVIEWER NAME**	Lim, Kuang Hock
**REVIEWER AFFILIATION**	Institute for Medical Research
**REVIEWER CONFLICT OF INTEREST**	none
**DATE REVIEW RETURNED**	09-Feb-2024

**GENERAL COMMENTS**	The manuscript is well written. However, the authors should carry out the minor correction as below:<br><br>Introduction:<br>More literature based on previous studies should be included to enhance the quality of the manuscript.<br><br>Methodology<br>The fit of Cox-Regression analysis should be described in the text.

VERSION 1 – AUTHOR RESPONSE

Reviewer: 1

Prof. Shizukiyo Ishikawa, Jichi Medical University

This is the population-based study in which title the study design was case-control study with 27 years follow up. The study was with long interval in which all inhabitants turning 40 or 50 years between 1990-1999 were invited for screening. However, the reviewer has some concerns about the present study.

Is the present study case control study?

Case-control study is the design which examine to compare the base-line exposure based on between cases and controls with outcome. However, the present study defined cases or controls as participants Mets or without Mets. The reviewer thought the present study was a kind of cohort study, but not typical.

This is a relevant question which we have discussed thoroughly with all authors. We concluded that it would be more correct to use the term “matched cohort- study” instead of case-control study. We are grateful for the reviewers comment and have changed to matched cohort-study in the title and throughout the manuscript. Accordingly, we have recalculated the hazard ratios and the corresponding 95% confidence intervals using stratified Cox regression model and revised the statistical analysis section. Page 6, line 1-5

Why whole cohort was not used?

Case-cohort study was thought more convenient than whole cohort study because of the cost or some other difficult condition to obtain many covariates from whole cohort subjects. However, the authors

used baseline data as total subjects of 34,269 (60.1%) people after screening by invitation and outcome of CV events and all-cause mortality from the index examination. The reviewer thought the whole cohort study was possible.

We understand the reviewers question regarding why the whole cohort was not used. Due to missing data for one or more co-variates this was not possible. The possibility to obtain missing data from the whole cohort is unfortunately not possible at this time (23-33 years later). Instead, we found matching individuals with MetS to two controls, with complete data on several important co-variates related to MetS, to be more appropriate.

Prevalence of Mets at baseline was not proper for the present study, because controls were 1:2 matched to cases. Hazard ratios calculated in cohort study were analyzed using whole cohort because of proper prevalence of risk factors as study samples. However, using matched controls for case-cohort study, proper hazard ratios were not calculated

We understand the reviewers concern regarding the design. As already written in the answer above we have concluded that matched cohort study better describes our study design. Accordingly, we have recalculated the hazard ratios and the corresponding 95% confidence intervals using stratified Cox regression models, where each stratum contains one MetS individual and two individuals matched one age, sex, and time of health examination. The Statistical Analysis section has been revised accordingly.

To clarify this, we have added the following information and one reference:

"Crude and adjusted prospective associations of MAP, MetS, and the factors retained from the factor analysis and first CV event and all-cause mortality were analysed for the total study population and by sex and age group separately, using stratified Cox proportional hazards regression models, where each stratum contains one MetS individual and two controls individually matched on age, sex, and health examination." Page 6, line 1-5

Added ref: Iwagami, M. and Shinozaki, T., Introduction to Matching in Case-Control and Cohort studies, Annals of Clinical Epimediology 2022;4(2):33-40.)

The study subjects were only 40 and 50 years old men and women. Distribution of the ages of the subject were limited in 40 and 50 years; 10 years apart. The results should be separately shown in 40 and 50 years, not all data to calculate risk ratios.

The reviewer was concerned that the situation around risk factors and outcome was changed or not for 10 years to obtain base line data.

This is a very relevant concern, and it is true that the situation regarding risk factors, drug treatment and time-trends for lifestyle habits may have shifted from the beginning of 1990 to the end of 1999. We aimed to address this time-dependent variation by matching individuals with MetS to controls who had their health examination the same year as often as possible. A small number of the controls had their health examination further apart, maximum five years. Second the stratification where each stratum contains one MetS individual and two individuals matched on age, sex, and time of inclusion will further enhance the analysis and provide a more robust analysis.

To clarify this, we have added the following wording under the paragraph Materials and methods, Study population

“Time of health examination was dichotomised to two groups, performed 1989 – 1994 or 1995-2000, with a vast majority of the matched controls having had their health examination the same year as the individual with MetS to whom they were matched.” Page 4, Line 15-17.

And under limitations, first paragraph:

“The screening for CVD risk factors may have induced pharmacological treatment, especially for the participants with the highest blood-pressure. As the data does not provide this information it is possible that pharmacological treatment may have had an impact on the results. In addition the situation regarding risk factors, drug treatment and time-trends for lifestyle habits may have shifted from the beginning of 1990 to the end of 1999. However, we aimed to address the time-dependent variation by stratification where each stratum contains one MetS individual and two individuals matched on age, sex, and time of inclusion” Page 9, Line 14-18

Reviewer: 2

Dr. Kuang Hock Lim, Institute for Medical Research

Introduction:

More literature based on previous studies should be included to enhance the quality of the manuscript.

We have completed the following sentence and added two more references; Kee Chee C et al 2021, and Faijer-Westerink et al, 2020 to further highlight the presence of MetS in different populations.

“Despite different definitions of MetS many studies with different populations have shown that when the criteria for MetS are reached the individual is at higher risk for cardiovascular disease (CVD), CVD-related mortality and all-cause mortality (4-9).” Page 3, Line 17

Methodology

The fit of Cox-Regression analysis should be described in the text.

This is a relevant question. As written in the answer to the first reviewer we have concluded that the term matched cohort study, where MetS is the exposure, better describes the design used. Thus, we have recalculated the hazard ratios and the corresponding 95% confidence intervals using stratified Cox regression models, where each stratum contains one MetS individual and two individuals matched one age, sex, and time of health examination. The proportion hazard assumption for the covariates were checked by visual inspection of the log[-log(cumulative survival)] for categorical variables, and by visual inspection of the Schoenfeld residuals versus time for continuous variables.

This is further elaborated in the Statistical Analysis section. Page 6 Line 1-9.

VERSION 2 – REVIEW

**REVIEWER NAME**	Ishikawa, Shizukiyo
**REVIEWER AFFILIATION**	Jichi Medical University, Department of General Medicine
**REVIEWER CONFLICT OF INTEREST**	none
**DATE REVIEW RETURNED**	12-Apr-2024

**GENERAL COMMENTS**	The reviewer thought matched cohort- study is more appropriate than case-control study for study design.<br><br>Table 1<br>Authors wrote “A total of 5084 individuals met the criteria for MetS were identified and were individually matched to two controls without MetS (n= 10 168) with regards to age, sex and date of health examination. Of the total study population (n=15 252)”.<br>The reviewer thought general characteristics in Table 1 for matched cohort study in this study, should be shown characteristics for between MetS and non-MetS, even if stratified analysis were made.

VERSION 2 – AUTHOR RESPONSE

Reviewer: 1
Prof. Shizukiyo Ishikawa, Jichi Medical University

Table 1
Authors wrote “A total of 5084 individuals met the criteria for MetS were identified and were individually matched to two controls without MetS (n= 10 168) with regards to age, sex and date of health examination. Of the total study population (n=15 252)”.
The reviewer thought general characteristics in Table 1 for matched cohort study in this study, should be shown characteristics for between MetS and non-MetS, even if stratified analysis were made.

We thank the reviewer for the feedback and have revised Table 1 in accordance with Reviewer 1´s recommendation, i.e., characteristics are shown for between MetS and non-MetS, for each age group and sex, respectively. Due to the formatting guidelines of BMJ Open this table has been designated to Supplemental material.

	Women, 40 years old (n=1 800)		Women, 50 years old (n=5 304)		Men, 40 years old (n=2 691)		Men, 50 years old (n=5 457)		Matched cohort population (n=15 252)	
	MetS (n=1200)	Non-MetS (n=600)	MetS (n=1768)	Non-MetS (n=3536)	MetS (n=897)	Non-MetS (n=1794)	MetS (n=1819)	Non-MetS (n=3638)	MetS (n=5084)	Non-MetS (n=10168)
Systolic blood pressure, mmHg	135 (130, 141.5)	120 (110, 130)	140 (130, 152)	126.5 (120, 140)	136 (130, 145)	125 (120, 135)	140 (130, 150)	130 (120, 140)	140 (130, 150)	125 (120, 140)
Diastolic blood pressure, mm Hg	85 (80, 90)	80 (70, 85)	90 (82, 95)	80 (75, 88)	89 (85, 95)	80 (75, 85)	90 (85, 95)	84 (80, 90)	90 (85, 95)	80 (75, 90)
Total Cholesterol, mmol/L	6.2 (5.4, 6.8)	5 (4.4, 5.6)	6.5 (6.0, 7.1)	5.5 (4.9, 6.1)	6.5 (6.1, 7.0)	5.3 (4.6, 6.0)	6.6 (6.1, 7.2)	5.6 (5.0, 6.3)	6.5 (6.1, 7.1)	5.4 (4.8, 6.1)
Mean arterial blood pressure (MAP), mmHg	10.3 (9.7, 10.7)	9.2 (8.7, 10.0)	10.6 (10.0, 11.3)	9.7 (9.0, 10.4)	10.3 (9.8, 11.0)	9.5 (9.0, 10.1)	10.7 (10.0, 11.3)	9.8 (9.3, 10.7)	10.5 (10.0, 11.2)	9.7 (9.0, 10.3)
Plasma glucose, mmol/L	5.9 (5.6, 6.5)	5.3 (4.8, 5.7)	5.9 (5.6, 6.4)	5.3 (4.9, 5.7)	5.9 (5.6, 6.6)	5.3 (4.9, 5.8)	6.0 (5.6, 6.7)	5.3 (4.9, 5.9)	5.9 (5.6, 6.6)	5.3 (4.9, 5.8)
Waist circumference, cm	92 (87, 100)	76 (72, 83)	92 (86, 99)	78 (73, 84)	103 (94, 108)	90 (85, 96)	102 (94, 107)	92 (87, 98)	97 (90, 105)	86 (78, 93)
Hip circumference, cm	109 (103, 116)	100 (96, 106)	109 (103, 115)	101 (97, 106)	107 (103, 111)	102 (99, 106)	107 (102, 111)	102.5 (99, 106)	108 (103, 113)	102 (98, 106)
Body Mass	28.7	23.4	28.8	24.2	28.6	24.9	28.3	25.4	28.6	24.7

Index, kg/m^2	(26.4, 32.4)	(21.9, 25.9)	(26.1, 32.0)	(22.3, 26.4)	(25.8, 31.0)	(23.1, 26.6)	(26.0, 30.7)	(23.7, 27.4)	(26.0, 31.3)	(22.8, 26.8)
Current smoker, n (%)	231 (38.5)	387 (32.3)	572 (32.4)	1054 (29.8)	270 (30.1)	462 (25.8)	521 (28.6)	982 (27)	1594 (31.4)	2885 (28.4)
Living alone, n (%)	43 (7.2)	55 (4.6)	186 (10.5)	409 (11.6)	142 (15.8)	244 (13.6)	228 (12.5)	445 (12.2)	599 (11.8)	1153 (11.3)
Lower education, n (%)	141 (23.5)	141 (11.8)	649 (36.7)	850 (24.0)	177 (19.7)	209 (11.6)	625 (34.4)	893 (24.5)	1592 (31.3)	2093 (20.6)

Supplemental Table 2. Baseline characteristics of study participants.

Variables are presented with median (Q1, Q3) or frequency and (percentage). MetS= Individuals that met three or more of the following risk factors were classified with metabolic syndrome (MetS): waist circumference: ≥102 cm (men) and ≥88 cm (women), total cholesterol: ≥6.1 mmol/ l, blood pressure: ≥130 and/ or ≥85 mm Hg (or previous diagnosis of hypertension), plasma glucose: ≥5.6 mmol/ l (or previous diagnosis of type 2 diabetes).
